# Nutritional status, body composition and diet quality in children with cancer

**DOI:** 10.3389/fonc.2024.1389657

**Published:** 2024-04-19

**Authors:** Magdalena Schab, Szymon Skoczen

**Affiliations:** ^1^ Doctoral School of Medical and Health Science, Jagiellonian University Medical College, Krakow, Poland; ^2^ Department of Pediatric Oncology and Hematology, Institute of Pediatrics, Faculty of Medicine, Jagiellonian University Medical College, Krakow, Poland

**Keywords:** childhood cancer, diet quality, body composition, nutritional status, dietary habits

## Abstract

During cancer treatment, nutritional status disorders such as malnutrition or obesity affect the tolerance of cancer treatment, quality of life, but also the pharmacokinetics of drugs. It is hypothesized that changes in fat and lean body mass can modify chemotherapy volume distribution, metabolism and clearance. In children with cancer, lean body mass decreases or remains low during treatment and fat mass increases. Body composition is influenced by the cancer itself, aggressive multimodal-therapies, changes in metabolism, unbalanced diet and reduced physical activity. Due to the side effects of treatment, including changes in the sense of taste and smell, nausea, vomiting, diarrhea, and stress, eating according to recommendation for macronutrients and micronutrients is difficult. Research indicates that throughout cancer treatment, the consumption of fruits, vegetables, and dairy products tends to be insufficient, whereas there is an elevated intake of sugar and unhealthy snacks. Children exhibit a preference for high-carbohydrate, salty, and strongly flavored products. This review revealed the importance of body composition and its changes during cancer treatment in children, as well as eating habits and diet quality.

## 1 Introduction

Nutritional status in patients with cancer is one of prognostic value affecting quality of life, drug metabolism and treatment tolerance (1). The rate of malnutrition in the population of children with cancer is ranging between 40–90% in lower-middle-income countries and between 0–30% in high-income countries (2). Overnutrition ranges between 8% and 78% (3). The consequences of nutritional disorders in cancer patients can be very serious. These include changes in pharmacokinetics and distribution of drugs, prolonged neutropenia, increased risk of infection and treatment toxicity, as well as extended duration of oncological treatment (4, 5). Weight loss greater than 5% body mass in the first 3 months of treatment and >10% after 6 months was associated with poorer survival. In patients with hematological malignances and solid tumors, weight loss greater than 20% increased the risk of death (6). Moreover, malnourished children had worse physical and social functioning while overweight had emotional and social problems (7).

To evaluate the nutritional status, clinical observations, anthropometric and biochemical measurements, nutritional indicators and questionnaires can be used (1). Each method has different sensitivity in assessing nutritional status disorders. Not all individuals with body weight falling within the normal range are adequately nourished. BMI does not distinguish between fat and muscle mass (8). A cost-effective and easily accessible method is the measurement of mid-upper arm circumference (MUAC), triceps skinfold thickness (TSFT), and arm muscle circumference (AMC), based on which the content of muscle and fat tissue can be estimated (1). Body composition assessment methods will be discussed in the following chapters.

Assessment of diet quality should also be part of the nutritional status control (1). Research shows that parents and caregivers of children with cancer have difficulties in composing a diet in accordance with the standards (9, 10). Children are more likely to eat high-carbohydrate (11), salty snacks and strong-flavor products (10), but they limit the consumption of vegetables and fruits (11).

In this review, we summarize changes in body composition and its role during treatment, the importance of diet quality and eating habits in children with cancer.

## 2 Nutritional status in children with cancer

The nutritional status of patients with cancer depends on many factors, such as the stage of the disease, type of cancer, nutrients intake, changes in metabolism and side effects of treatment. There is also noticeable increased secretion of pro-inflammatory cytokines such as IL-1, IL-6, IFN-γ, TNF-α (IL-1—interleukin 1, IL-6—interleukin 6, INF-y—interferon gamma, TNF-α - tumor necrosis-α) and substances secreted by the tumor such as PIF, PMF, LMF (PIF-protein inducing factor, PMF—protein mobilizing factor, LMF-lipid mobilizing factor) which cause changes in metabolism such as increased lipolysis, proteolysis, glycolysis (2). Patients on high-risk treatment protocols are more likely to be malnourished (12). In contrast, there are also drugs such as corticosteroids, which increase the risk of overweight and obesity. Undernutrition, overnutrition, and obesity are associated with adverse outcomes from diagnosis to survival (2).

Leukemia is the most common type of cancer in children. The prevalence of malnutrition ranges from 5-10% at diagnosis, while the prevalence of overnutrition ranges from 2.9% in acute lymphoblastic leukemia (ALL) to 14.9% in patients with acute myeloid leukemia (AML) (5). Studies show that higher BMI at diagnosis is associated with worse overall survival (OS), event-free survival (EFS), and increased mortality in children both with AML and ALL (13–16). Children with decrease in BMI during first 32 weeks of treatment had poorer OS [hazard ratio (HR): 2,10, 95% confidence interval (CI) (1, 14–3, 87)] than children without loss in BMI. Moreover, in children with ALL, both undernutrition and overnutrition were associated with higher risk of relapse (17–19). The importance of nutritional status in other hematologic malignancies is not well understood but has been shown that in children with Hodgkin’s lymphoma, there is an association between malnutrition and worse OS (17). In many studies lymphomas are grouped with leukemias, and common conclusions are drawn.

Summary of nutritional status assessment in children with solid tumors is difficult due to the limited number of studies and different assessment methods used (2). It is believed that children with solid tumors have higher risk of malnutrition than children with hematological malignances (20). In this group of patients due to mass of the tumor, the appropriate assessment method is the triceps skinfold thickness (TSFT) and mid-upper arm circumference (MUAC) rather than weight-for-height (W/H) index and body mass index (BMI) (21). Unfortunately, much of the research conducted so far has been based on these indicators. It has been shown that in children with solid tumors, an abnormal BMI was associated with worse response to treatment. Moreover, low BMI was linked with increased risk of cardiotoxicity, wound infection, and worse OS, while high BMI was associated with higher risk of arterial thrombosis, nephrotoxicity, worse OS and EFS (17, 22–26).

Children with brain tumors are also a group with insufficient data on the assessment of nutritional status. It is believed that these patients are especially predisposed to overweight and obesity compared to children with other types of cancer (12). Tsutsumi et al. has shown that at diagnose almost a quarter (23,3%) of children with brain tumor was overweight (12). According to Diakatou & Vassilakou the rates of overnutrition and obesity can be even higher - 42.6% and 40.4% respectively (2). Peng et al. indicated that children with WHO grade 1–2 brain tumor had higher BMI, waist circumference and TSFT compared to patients with WHO grade 3–4 brain tumor. Moreover, in this group of patients increased blood pressure was associated with Nuchal Skinfold Thickness (NST), BMI and waist-to-height ratio (27). It is worth noting that although previous authors indicated an increased risk of overweight and obesity, Brinksma et al. showed that children with brain tumors has lower fat free mass compared to children with hematological malignancies and solid tumors (28).

Patients from the adolescent and young adult (AYA) cancer population are also at risk of nutritional status disorders during and after oncological treatment (29). In a study conducted by van der Haak N. et al. it was observed that the percentage of underweight patients increased during treatment (8% vs. 20% (p = 0,012)), and that 44% of patients lost >5% of body weight during treatment. A relationship between the type of cancer and the risk of weight loss has also been demonstrated. Eighty six percent of patients with ALL/lymphoblastic lymphoma (LL) and AML experienced ≥5% loss of weight during treatment compared with 17% with Hodgkin lymphoma (p < 0,0001). During survivorship, patients with leukemia and lymphoma had an increased risk of being overweight and obesity compared to other diagnoses (67% vs. 14%, p = 0,037) (29).

In adult patients, nutritional status disorders during and after treatment are also common. Malnutrition occurs in 31-87% of adult patients at the time of diagnosis, depending on the type of cancer, stage of disease and individual patient characteristics. Furthermore, weight loss at diagnosis was associated with shorter failure-free and worse overall survival (OS) (30). Muscaritoli M. et al. conducted a prospective study and observed that 51,1% of all cancer patients had nutritional disorders, and 64% of patients showed decrease in weight 6 months after diagnosis (31). Petrelli F. et al. published a meta-analysis involving over 6.3 million patients with cancer, and observed that obese patients with breast, colorectal and uterine cancer had increased overall and cancer-specific mortality. However, in patients with renal cell carcinoma, lung cancer, or melanoma, better survival than patients without obesity was observed (32).

The above data indicate that nutritional disorders in children undergoing cancer treatment are common and their consequences are serious. It is important to look for the causes and methods of preventing nutritional disorders. Changes in body composition during treatment and diet quality in pediatric oncology patients should be analyzed.

## 3 Body composition in children with cancer

### 3.1 Body composition assessment methods

The role of changes in body composition during cancer treatment is increasingly discussed (33). To assess the content of lean and fat body mass, an appropriately sensitive measurement method should be selected. One of the well-known method is dual-energy X-ray absorptiometry (DXA), which involves weakening the beam of ionizing radiation passing through tissues of various densities. This method is relatively cheap and quick to perform but has limited use in everyday practice (34). DXA can only indicate regional differences in body composition in 2-dimensions and does not distinguish visceral from subcutaneous fat (5, 34). Other methods include bioelectrical impedance test (BIA), which among others estimate fat, muscle and water mass using impedance of electrical current (5). It is important to choose an analyzer with several current frequencies, which increases the accuracy of the result (1). This method avoids radiation exposure but has limited use in chronically ill patients and those with edema (5). Anthropometric measurements of the arm such as MUAC, TSFT, and arm muscle circumference (AMC), are a cheap and easily available method to estimate the content of muscle and fat tissues (1, 35). Other techniques for assessing body composition include air displacement plethysmography (ADP). It is non-invasive and cheap method, but the results can be altered by the patients movements and body temperature. Moreover, it is impossible to show regional changes in body composition using ADP method (34). Computed tomography (CT) and magnetic resonance imaging (MRI) can also be used to estimate body composition. These techniques are precise and capable of discerning between lean body mass, subcutaneous fat, and visceral fat, however they are expensive and expose to radiation in case of CT (5, 36). It is believed that multi-slice CT images provides the most precise measurement of various tissue compartment, however single-slice CT image at L3 vertebral level is increasingly used body composition assessment method in many studies (36). The table below summarizes studies comparing various methods of assessing the nutritional status of children with cancer (**Table 1**).

**Table 1:** Summary of studies assessing nutritional status in children with cancer using various methods.

Reference	Assessment method	Type of cancer	Patients number (n)	Age [years]	Outcomes
(37)	D2O dilution, BIA, body weight, height, circumferences, skinfold measurement	Hematologic malignancies, solid tumors	N= 14	5.6 – 13.6	• Good correlation between BIA and D2O - no significant difference in the measurement of FFM and FM • Higher TBW (%) estimated by the BIA than by the D2O dilution • Anthropometric measures correlated with D2O
(8)	Body height/length, weight, MUAC, TSFT, AMC, BMI, albumin level, history of weight loss at diagnosis	mix	N=17	2-15	• The prevalence of malnutrition were: BMI 38%, TSFT 57%, MUAC 76%, AMC 69%, TSFT + MUAC 81% • Adding BMI to arm anthropometry increased the percentage of severely malnourished patients by 2% and serum albumin by 1.5% • Significant weight loss identified 16.5% of individuals at nutritional risk overarm anthropometry
(38)	DXA, MUAC, TSFT, BMI	mix	99	< 20	• Good correlation between MUAC and LBM (0,90), • Poor correlation between TSFT and FM (0,70)
(39)	Body weight, height, BMI, MUAC, TSFT, serum albumin level, prealbumin level	mix	81	0,2-17,3	• The prevalence of malnutrition were: BMI 23.5%, MUAC 27.2%, TSFT 21% • Correlation between BMI, MUAC and TSFT was moderate at initial diagnosis and higher in the follow up • Correlation between MUAC and TSFT was high both at initial diagnosis and in the follow up • The concentration of serum albumin did not allow the identification of malnutrition detected using anthropometric methods
(40)	DXA, MUAC, TSFT, serum albumin	Hematological malignancy, solid tumors	137	10.5 (+/- SD = 4.2)	• The prevalence of malnutrition were: BMI 14%, TSFT 23%, MUAC 45%, AMC 64% • MUAC was the best predictor of undernutrition with lowest standard error
(41)	BMI, MUAC, AMC, arm fat area	Children undergoing allogeneic HCT for a hematologic malignancy	733	2-18	• AMC <5 percentile appears to be a stronger predictor of poor HCT outcomes than BMI
(42)	Anthropometric data, BIA, REE	ALL	15	2.17-12.2	• Good correlation between FFM obtained by anthropometry and by BIA

D2O dilution, deuterium oxide dilution; BIA, bioelectrical impedance test; FM, fat mass; FFM, fat free mass; TBW, total body water; AMC, arm muscle circumference; DXA, dual-energy X-ray absorptiometry; MUAC, mid-upper arm circumference; TSFT, triceps skinfold thickness; LBM, lean body mass; REE, resting energy expenditure; HCT, hematopoietic cell transplantation; ALL, acute lymphoblastic leukemia; SD, standard deviation.

### 3.2 Body composition changes during cancer treatment

Changes in body composition are caused primarily by cancer itself, aggressive multimodal-therapies, changes in metabolism, unbalanced diet and reduced physical activity. It was indicated that in pediatric cancer patients the decrease in lean body mass was associated with the length of hospitalization (r=0,31, P<0.05) and the burden of illness (43). Higher skeletal muscle density (SMD) was correlated with lower grade hematologic toxicities in children with lymphoma and rhabdomyosarcoma (44). Still little is known about body composition changes during treatment. The research conducted so far has focused mainly on anthropometric measurements and BMI.

It has been shown that the body composition of children newly diagnosed with cancer does not differ significantly from that of healthy children. It is believed that the greatest changes in body composition occur in the first months of treatment. Revuelta Iniesta et al. observed that during the first 3 months of therapy as fat mass increased, fat-free mass decreased and this trend persisted until the end of the study (36 months) (12). Similar results were obtained by Brinksma et al. They showed that in the first 3 months of treatment, fat mass and BMI increased, while fat free mass was low at diagnosis and remain low to the end of the study (28). Yang and Choi observed that in the group of children with hematological malignances (n=19) and solid tumors (n=11) the percentage of body fat increased during first months of treatment, but no differences were found between measurements after 1 and 12 months of treatment. At the same time, the fat-free mass was reduced in the first months of treatment and then increased within 6-12 months (45). The increase in fat tissue is particularly noticeable in children with hematological malignancies (37). Murphy et al. and Brouwer et al. confirm that children undergoing cancer therapy had higher percentage of body fat, fat mass index, lower cell mass index and fat free mass index compared to healthy ones (46–48). Importantly, muscle loss and fat gain during cancer treatment can occur despite eating below recommended calorie levels and increasing protein intake (49).

Halton JM. et al. observed decrease in height standard deviation (SD) score in children with ALL in the first year of treatment and a further decrease in children who received cranial irradiation (50). Moreover, they observed loss of weight in the first year of treatment, and after this time children had tendency to disproportionate weight gain. The mean body fat mass increased from 22% to 28% after finishing therapy. Children in remission from ALL had higher BMI and fat mass compared with healthy control group (51).

AYA cancer brain tumors survivors and cranial radiotherapy patients experience changes in body composition and deficits in muscular strength (p=0,009), muscular endurance (p=0,30) after treatment. Survivors had lower lean body mass (p = 0,004), bone mineral density (p = 0,005), and higher percentage of total body fat (p = 0,017), central fat (p = 0,009) and peripheral fat (p = 0,032) (52). Wooten S.V. observed significant reductions in total skeletal muscle index (p < 0.01) and density (p=0,04) early after chemotherapy in AYA cancer patients (53).

Available data indicate significant changes in body composition during oncological treatment in children. The most common changes were a decrease or low level of lean body mass and an increase in fat mass.

### 3.3 Sarcopenia

**Figure 1:**
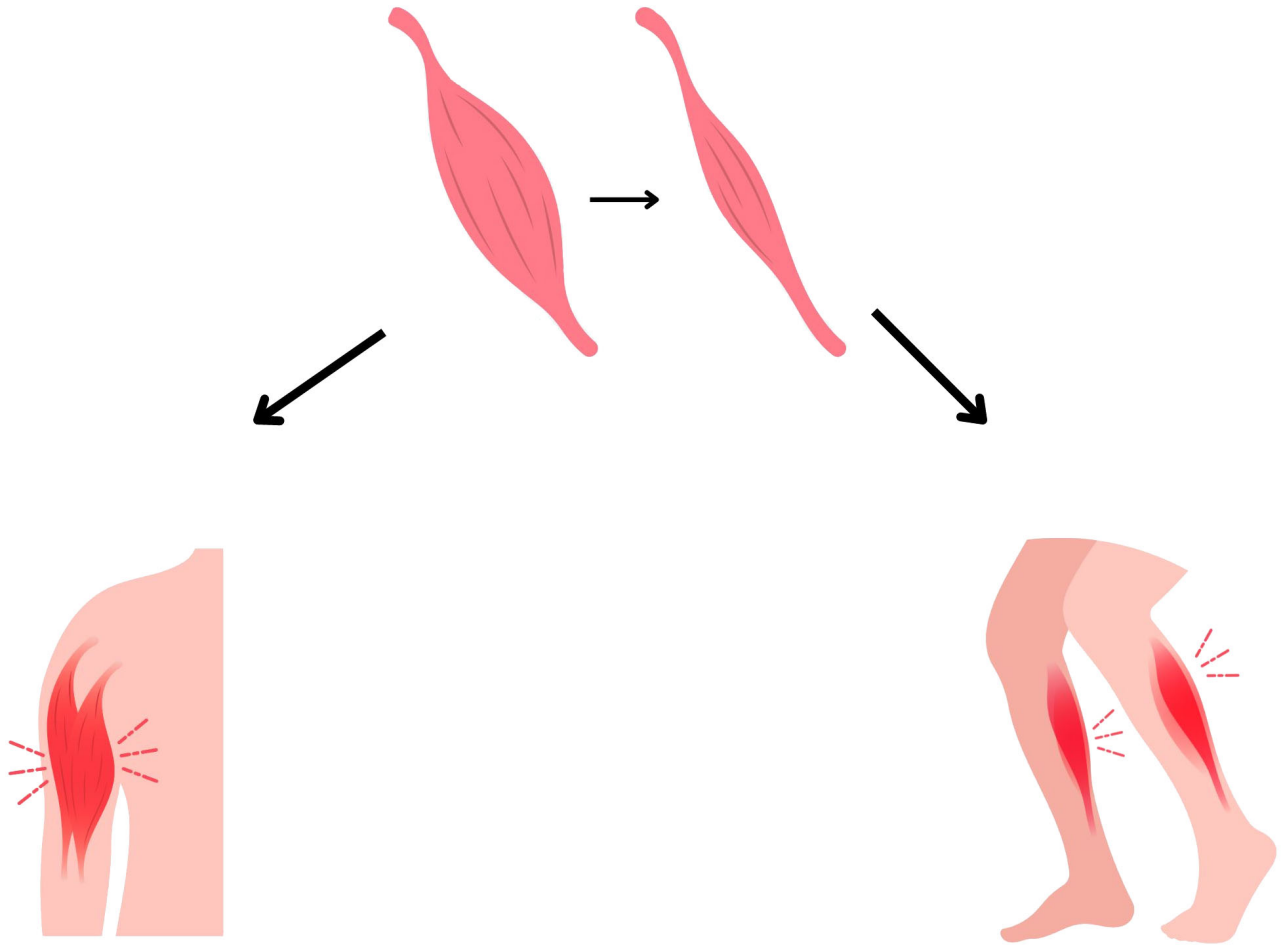
Muscle loss in sarcopenia.

Sarcopenia is defined as skeletal muscle wasting (**Figure 1**). It may coexist with normal, depleted or excess fat body mass. It is also one of the prognostic indicators in adult oncology patients (36). Sarcopenia in oncological patients has very severe long-term consequences. It has been shown, that sarcopenic patients has higher risk of postoperative complications, infection, longer hospital stays, poorer treatment tolerance and more common dose-limiting toxicities (54, 55).

In children it is most noticeable in ALL patients both undergoing treatment (56) and in long-term survivors (57). Rayar et al. observed that children with ALL experience significant loss of skeletal muscle mass during first 6 months of treatment (43). Suzuki D. et al. conducted a study using CT imaging at the L3 level in children with ALL. It was observed that all participants experienced a loss of skeletal muscle mass after induction, and 25% of children developed sarcopenia. Moreover, sarcopenia was associated with serious adverse events (p=0,09) and invasive fungal infection (p=0,018) (58). In children with ALL loss muscle mass often coexists with excessive fat mass defined as sarcopenic obesity. It is also common in survivors of hematopoietic stem cells transplantation (HSCT) and after total body irradiation (TBI) (2). Fuemmeller et al. observed that in the first year of treatment, children with ALL and lymphoma had higher BMI, fat mass and lower percentage of lean body mass at baseline and after 12 months, compared with healthy control (59). In recent years, sarcopenia has been increasingly observed in patients with solid tumors (60). Tostes et al. showed that muscle strength is associated with nutritional status and muscle mass (61). In children with cancer handgrip strength (HGS) was strongly positive correlated with mid-arm muscle circumference (MAMC) and body weight (r=0,743, p < 0,001 and r=0,706, p < 0,001, respectively). Moreover, the lowest quartile of HGS was associated with MAMC below the adequate level (p = 0,005) (61).

Details about research on sarcopenia and muscle mass are listed in **Table 2**.

**Table 2:** Summary of research on sarcopenia and muscle mass.

Year, Reference	Type of cancer	Patients number (n)	Age [years]	Assessment method	Outcomes
2024 (62),	ALL survivors	74	21,0 (range: 13,5–38,3),	pQCT - CSA	• sarcopenic obesity was observed in 32 (44,4%) patients • CSA Z scores may be a useful clinical measure of SMM
2023 (63),	Childhood cancer survivors	3996	18–45	DXA - lean mass of the arms and legs	• in childhood cancer survivors, frailty and sarcopenia occur already at a mean age of 33 years
2023 (64),	Wilms tumor	38	1-14	CT - TPMA	• at diagnosis, 55,3% of patients had sarcopenia, after 4–6 weeks of neoadjuvant chemotherapy 73,7%, this rate remained high (78,9%) for 1 year • using TPMA/age Z-score significant and rapid muscle loss was observed, with little or no recovery in the study period
2023 (65),	Ewing sarcoma (n = 34) osteosarcoma (n = 26)	60	Mean 13 (range 1,5–18)	CT - total muscle areas of the pectoralis, paraspinal (T12 level) and psoas (L4 level) muscles and total abdominal muscle area (L3 level) (within 1 month of the initiation of chemotherapy)	• No skeletal muscle index or sarcopenic obesity index (calculated by dividing SMI by body mass index) parameter significantly affected event-free or overall survival in the total group analysis • in the non-metastatic group, higher values of SMI–paraspinal and SMIT12– psoas were correlated with longer EFS
2022 (66),	Ewing sarcoma Rhabdomyosarcoma Desmoplastic tumor	21	median 10,5 (IQR 6,6, 15,1)	CT - tPMA detected by axial CT images of the L4–L5 vertebrae, at diagnosis and after 12 months of treatment	• sarcopenia was observed in more than half (57,1%) of patients at diagnosis • reduction of tPMA z-score between diagnosis and after 12 months of treatment (p < 0,05)
2021 (67),	Hepatoblastoma	33	median 2,15 (IQR 1,47, 3,24)	CT, MRI - tPMA was measured at intervertebral disc levels L3-4 and L4-5	• sarcopenia was observed in 52% patients • poor correlation between tPMA and weight • children with high risk of relapse had sarcopenia before surgery • after surgery, tPMA z-score did not improved in patients with relapse, while tPMA z-score improved in 75% patients without relapse
2021 (68),	High risk neuroblastoma	29	median 3.0 (IQR 2,0–4,5)	CT - cross-sectional area of skeletal muscle, skeletal muscle density at L3	• increase in skeletal muscle (p = 0,029), skeletal muscle density (p = 0,002), intermuscular adipose tissue (p < 0,001) was observed
2021 (69),	Neuroblastoma	101	median 3 (IQR 2,25–5)	CT, MRI - tPMA measured at lumbar disc levels L3-4 and L4-5	• children with sarcopenia had decreased 5-year survival • no association between sarcopenia and short-term outcome • moderate correlation between tPNA z-score at L4-5 with weight-for-age z-score
2021 (70),	High risk neuroblastoma	20	mean 12.4 (SD 1,6)	DXA - leg lean mass, appendicular lean mass and total body lean mass	• high risk neuroblastoma survivors had lower leg lean mass (p<0,001) and strength (p<0,001) compared with controls
2021 (71),	High risk neuroblastoma	24	median 2 (range 0–6)	CT – PMI at L4 at 4 time points	• prominent PMI reduction was observed between diagnose and after first cycle of chemotherapy (7,09 ± 0,99 vs. 6,01 ± 0,98, P < 0,001) • younger age and male sex may were predictive factors for PMI recovery
2021 (72),	ALL survivors	65	15 (7,7–27,5)	DXA - LBM	• significantly higher prevalence of sarcopenic obesity identified by DEXA (14%) among survivors as compared to the controls (0/50, P<0,001), • similar prevalence of sarcopenia detected by LBM 60% (39/65) in survivors group and in control group 56% (28/50)
2021 (73),	long-term survivors of childhood leukemia/lymphoma	81	25 (18–53)	BIA	• Sarcopenia was observed in 7 (21%) of 33 survivors with HSCT and 2 (4%) of 48 survivors without HSCT (P = 0,012).
2020 (74),	Ewing sarcoma, Osteosarcoma, Rhabdomyosarcoma, Wilms tumor	39	median 11 (range 1,33–20)	CT - SM at intervertebral level T12-L1 (n = 39), L3 (n = 22)	• significant decrease in SM (−4,2 ± 8,12, p = 0,003) and RLT (−10,7 ± 28,5, p = 0,025) was observed after 6-14 weeks after initiation of therapy
2019 (75),	High risk neuroblastoma	13	progression-free survival group: mean 2 (range 0–5) relapse/death group: mean 3,1 (range 2–5)	CT - PMA of the L3-level lumbar spine	• patients with rate of change in the PMA >1,00 showed a prolonged overall (P =0,0078) and progression-free survival (P=0,006) (The rate of change was calculated by comparing the PMA of the L3-level lumbar spine on CT before and after treatment with the standard protocol)
2017 (76),	High risk neuroblastoma	19	median 22 (range 16–30) 33,1 years (SD 7,2)	DXA - whole body LMI	• neuroblastoma survivors had significantly lower body lean mass adjusted for height (P = 0,001)
2017 (58),	ALL	47	Median 8,6 (0,9 – 16)	CT - PMA	• Decrease in muscle loss in all patients after induction therapy • sarcopenia was associated with serious adverse events (p=0,09) and invasive fungal infection (p=0,018)
2017 (77),	ALL, lymphoma	15	10,3 (4-118)	DXA – lean body mass (at three times point – baseline, after 6 and 12 months)	• children with cancer had significantly higher %BF compared to control at baseline and after 12 months (30,3% vs. 24,9%) and significantly less percent LBM than controls (6,3 kg less at baseline and 5,2 kg less) at 12 months
2016 (78),	ALL survivors	365	median age 28,5 years, range 23,6–31,7	DXA - total fat-free mass	• LMM [kg] and %LMM were lower in survivors group compared to controls • lean muscle mass relative to height did not differ between survivors and controls
2016 (56),	ALL	50	Mean 14,7 (9,9-19,6)	DXA - LMM at diagnosis, end of Induction, and end of Delayed Intensification	• the weight loss during induction was due to a significant decrease in LMM • significant changes in lean body mass [kg]– 41,6 ± 1,6, 35,6 ± 1,6, 36,4 ± 1,6 (p <0,001)
2012 (43),	ALL	91	6,1 years (median, 5,0, range 1,2 to 17,6)	DXA - SMM	• notable loss of SMM early in treatment was observed, with incomplete recovery • the degree of SMM loss was associated with the burden of illness and duration of hospitalization
1990 (79),	ALL	14	3,6 to 13,9 years of age	A muscle index was calculated from the femoral quadriceps muscle thickness, measured by using an ultrasound method, and from the body surface area.	• the highest degree of muscle loss developed by 4 to 6 weeks with an average of 27% decrease of the muscle index • regeneration of muscle mass occurred over the next 6 months

ALL, acute lymphoblastic leukemia; PMA, psoas muscle area; tPMA, total psoas muscle area; pQCT, quantitative computed tomography; CSA, calf muscle cross-sectional area; EFS, event-free survival; LBM, lean body mass; DXA – dual-energy X-ray absorptiometry; SMI, skeletal muscle mass index; CT, computed tomography; MRI, magnetic resonance imaging; IQR, interquartile range; PMI, psoas muscle index; BIA – bioelectrical impedance test; BF – body fat; LMM – low muscle mass.

In adult patients it was observed that in patients with solid tumors, pretreatment skeletal muscle index was associated with worse OS regardless the type and stage of cancer (HR = 1,44, 95%, CI =1,32 to 1,56; p<0,001) [Shachar et al., (80)]. In other studies sarcopenia was associated with worse functional status (81).

Some drugs used during cancer treatment cause impaired protein synthesis, increased protein degradation and adverse effects on the neuromuscular system (34). Neurotoxic chemotherapy like vincristine and methotrexate affects the neuromuscular system in many ways. One of the side effects of vincristine is peripheral neuropathy, manifested by distal muscle weakness, absent reflexes, and impaired flexibility (34). Methotrexate is a cytostatic drug that impairs the functioning of the nervous system in the context of motor recruitment and muscle strength, and consequently muscle growth (34). Acute neuropathy is diagnosed in 20-60% of children with ALL. In the case of lymphoma and non-central nervous system (CNS) solid tumors, this value increases to 85% of patients (82). Symptoms of neuropathy may persist during and after cancer treatment (82). It can be suspected that neuropathy occurring simultaneously with sarcopenia exacerbates the problem and complicates the rebuilding of muscle tissue. L-asparaginase also impairs muscle health in children with cancer and survivors (34). This drug inhibits muscle protein synthesis and metabolizes glutamine, which is an amino acid essential in the process of protein synthesis and breakdown (83).

Corticosteroids are another group of drugs used in cancer treatment that impair muscle function. They cause muscle atrophy by increasing muscle breakdown and impairing the protein synthesis process (34), which can also exacerbate sarcopenia.

### 3.4 The influence of changes in body composition on the pharmacokinetics of drugs

The mechanism underlying the association between body composition and health outcomes in cancer patients is not fully understood. It is hypothesized that changes in fat and lean tissue mass affect chemotherapy volume distribution, metabolism and clearance from systemic circulation (58). Protein deficiencies and decreased protein intake may reduce renal blood flow and glomerular filtration, as well as renal tubular secretion (84, 85). It is increasingly noticed that dosing drugs into body surface area (BSA) does not predict drug pharmacokinetics and that BSA has poor correlation with fat-free mass (81).

There are only few studies in the literature assessing the pharmacokinetics of drugs in pediatric oncology depending on nutritional status. A systematic review on this topic, including 4 studies showed that undernutrition can alter pharmacokinetics of vincristine. In the analysis, 668 children were included, of whom 121 (18%) were malnourished and exhibited a significantly reduced vincristine clearance (86).

Thompson et al. observed that doxorubicin clearance was decreased in children with body fat percentage above 30%. They suppose that this can be one of the factors increasing the risk of treatment-related cardiac toxicity in this group of patients (87). Children with solid tumors and obesity at diagnosis treated with cisplatin, has three times higher risk of treatment related toxicity (p=0,037), especially manifested as acute or chronic kidney damage (p=0,014) (88).

In contrast, Hijiya et al. did not observed influence of BMI on pharmacokinetics in 621 children with ALL (89). BMI does not distinguish between fat mass and lean tissue mass, which may be one of the reasons for the lack of correlation.

Studies in adults have shown that methotrexate clearance was reduced, and the elimination time was prolonged in malnourished patients compared to well-nourished patients (90, 91). Reduced vincristine clearance was also observed in malnourished patients with Wilms tumors (92). An association between body composition and TXT treatment with immunotherapeutic agents has also been described in adult patients (58).

Animal studies indicate that the pharmacokinetics of doxorubicin and 5-fluorouracil may be altered by protein deficiencies (93, 94).

Available data allow to suspect the existence of a relationship between body composition, changes in drug pharmacokinetics and the related toxicity of treatment in children with cancer.

### 3.5 Interventions to prevent sarcopenia and changes in body composition

Preventing sarcopenia in pediatric cancer patients poses a formidable challenge. It is very important to look for new ways to prevent the loss of lean body mass. Barbosa-Cortés et al. conducted randomized control trial in children newly diagnosed with ALL supplemented with long-chain polyunsaturated fatty acids (LCPUFA) - 0,100 g/kg of body weight/day for 3 months. Body composition was measured by DXA at diagnosis, remission and 3 months after supplementation. In the study group, the decrease in lean body mass (LBM) was smaller than in the control group in remission (p = 0,044) and after 3 months of supplementation (p = 0,039). Lean body mass at remission was associated with higher DHA (r = 0,487, p = 0,034) and EPA (r = 0,499, p = 0,030) concentration in erythrocytes in the supplemented group (49). Morales J.S. et al. conducted the randomized controlled trial (NCT01645436), in which 49 children with cancer were divided into an intervention group and a control group. Children from intervention group (n = 24, 10 ± 4 years) performed 3 weekly training sessions (aerobic + strength exercises) by 19 ± 8 weeks (from the start to the end of neoadjuvant chemotherapy treatment). Authors observed improvements in all strength tests when compared mean values before and after intervention (p<0,001), with an overall positive response in seated bench press (80%), lateral row (88%), and leg press (93%). There was no significant improvement in functional mobility (assessed by the 3-meter Timed Up and Go (TUG) and Timed Up and Down Stairs (TUDS) tests) and cardiorespiratory fitness (CRF) (95). Braam K.I. conducted a study on a group of 68 children (26 children with intervention and 33 children from control group) with cancer whose intervention consisted of combined physical and psychosocial training. The intervention consisted of 24 individual sessions with a physiotherapist and 6 psychosocial sessions for the child and 2 for parents. The intervention lasted 12 weeks. Physical function was assessed at baseline, after 12 weeks and after 12 months. They observed improvements in lower body muscle strength in intervention group after 12-months when compared to the control group. No other significant differences were found (96). In 2016 Fiuza-Luces et al. carried out a clinical trial in children with solid tumor (n=24) to determine the effects of an inhospital exercise intervention combining aerobic and muscle strength training. Participants exercised 3 times a week (60-70 minutes per session) for 19 +/- 2 weeks. The intervention significantly increased muscle strength and performance after training: leg press: 40% [95% confidence interval [CI], 15–41 kg), bench press: 24%m [95% CI, 6–14 kg], lateral row 25% [95% CI, 6–15 kg]) (97). In 2020 Stössel S. et al. conducted a randomized controlled MUCKI trial in a group of children with cancer (n=16, mixed diagnoses) undergoing intensive cancer treatment, in which they used an intervention consisting of 45-60 minute exercise sessions for 6-8 weeks. They observed positive effects for leg strength, walking performance, fatigue, self-esteem (98). Manchola-González et al. also carried out a randomized clinical trial, in a group of 24 survivors of ALL (intervention group = 12, control group = 12). Changes in mean values were observed after intervention compared with baseline for Timed Up& Go test TUG (s) (P = 0.010), and Timed Up and Down Stairs test (TUDS s) (P = 0.001) (99). In 2022 Qing Shi et al. published systematic review and meta-analysis, which revealed that muscle strength improves in children with cancer after exercise intervention (n = 5 studies, n = 300 participants, (standardized mean difference (SMD) = 1,42, 95% CI = 0,10~2,74, p = 0,03) (100).

A randomized clinical trial titled “Pediatric Oncology Nutrition Intervention Trial (POINT)” is being conducted at the University of Kentucky. They are recruiting 45 newly diagnosed children with cancer, in whom they will assess changes in body composition and nutritional status in the context of intervention using oral nutrition supplements (ONS), appetite stimulants, and enteral nutrition (EN). Researchers will also assess the level of biomarkers (leptin, IL-6, Lipid profiles, Cystatin-C, Vitamin D, and C-reactive protein), compliance and tolerance to nutritional intervention, physical activity, and clinical outcomes. At the time of diagnosis, patients will receive standard medical nutrition therapy regarding high protein and high calorie diet. Nutritional assessment will include a 24-hour food recall. For the intervention group, measurements will be conducted at randomization and 1 and 3 months (101).

Another current ongoing study on body composition in children with cancer is the study conducted at the Universidad de Sonora entitled “Body Composition and Nutritional Status in Pediatric Patients With Hematological Malignancies (HM)”. To study will be enrolled 38 children with HM, whose body composition will be assessed at baseline and after 6 months using the deuterium dilution technique and electrical bioimpedance, as well as anthropometric indicators (102).

Benjamin-Eze J. is conducting a clinical trial on UNC Lineberger Comprehensive Cancer Center titled “Measuring Changes in Body Composition and Physical Function in Patients With Childhood Cancers”. They will include 30 children in the study and will assess BIA, D3-Creatine Dilution, 6-Minute Walk Test (6MWT), Timed Up and Go (TUG), 30-second Sit-to-Stand (STS), Hand Grip Strength (GS), PBTL p16 expression, CT, MR and PET Imaging. Assessments will be performed at diagnosis, once during active treatment, and end of treatment (103).

Sarcopenia is associated with many undesirable side effects. Additionally, many drugs can increase muscle loss and inhibit protein synthesis. Muscle strength can be improved through exercise intervention. It is necessary to conduct research on this topic in a group of children with cancer with various diagnoses.

## 4 Diet quality in children with cancer

Proper nutrition is essential for children’s growth, development and to obtain adequate bone and muscle mass (104). It is important both for children undergoing oncological therapy and for those who have completed treatment. Clark E. et al. showed that childhood cancer survivors require nutritional intervention in such aspect as picky eating, restricted and insufficient diet, poor quality diet, difficulties with eating after tube feeding, problem with weight gain (105). Furthermore, adult survivors of childhood cancer have increased risk of developing metabolic syndrome, obesity, osteoporosis, and cardiovascular disease (104). They have insufficient intake of calcium and folate (11, 106).

During cancer treatment, due to side effects such as vomiting, nausea, diarrhea, changes in the sense of taste and smell, as well as due to stress, the intake of macro- and microelements in recommended amounts is difficult. In the study conducted by Clark E. et al. it has been shown that 86% parents of cancer children had concerns about nutrition during treatment such as vomiting, anorexia and weight loss (9). In addition, it is a common practice among parents to increase the calorie content of diet by giving their child unhealthy snacks with low nutritional value (104). Parents also reported gaps in the current nutritional support system for patients in hospitals and the need for more accessible nutritional support (9). Napartuk M. et al. observed that one-year nutritional intervention introduced after diagnose can significantly improve the diet of children with cancer reflected by the Diet Quality Index. An increase in the mean z-scores for weight, BMI, mean levels of HDL-C and 25-hydroxy vitamin D was observed (107).

Cohen J. et al. showed that food intake by children undergoing cancer treatment is of reasonable quantity but of poor quality (108). It is worth noting that among oncology patients, still one of the recommended diets is a low-bacterial diet. A systematic review showed that it does not reduce the risk of infection and mortality, but it reduces quality of life and nutritional status (109). The following sections summarize the intake of nutrients and food groups in children with cancer.

### 4.1 Vegetables and fruits

Cohen et al. observed that most children undergoing cancer therapy did not eat enough vegetables (94%) and fruits (77%) (108). Soliman Bahgat et al. indicated that only 30% of children during treatment consume fruits and vegetables (110). Other authors confirm reduced consumption of products from this group during (77, 111) and after treatment (11).

### 4.2 Milk and dairy

Cohen et al. observed that the consumption of milk and milk alternatives in children with cancer was insufficient in 77% and 75%, respectively (108). Soliman Bahgat et al. indicated that only 30% of children undergoing cancer treatment eat dairy products (110). Galati et al. also observed low consumption of milk and dairy products (111).

### 4.3 Meat

Meat consumption varied depending on the study. Solinman Bughad et al. observed that after 3 months of treatment the percentage of children eating meat decreased (50% vs 21,67%) (110). Skolin et al. indicated the avoidance of meat in children with cancer (112). In contrast, Rohr et al. demonstrated increased desire for meat in children with ALL during induction and reinduction (113). Also Galati et al. observed that children with cancer had a higher intake of meat compared to the recommended amount (111).

### 4.4 Micronutrients and vitamins

Galati et al. observed that children with cancer had lower intakes of zinc, phosphorus, riboflavin, vitamin B12 and higher intakes of potassium compared to control group (111) Tan et el. indicated that calcium intake in children with cancer was below recommendations (114). Soliman Bahgat et al. compared the intake of calcium, iron, vitamins A and C at the first contact and after 3 months of treatment and did not observe significant changes (110). Tah et al. indicated that children with hematology malignancies have higher intake of vitamin A and B3 compared to children with solid tumors (108). Moreover, Cohen et al. observed that 65% of children with cancer exceeded recommendation for sodium (108). However, Bélanger V et al. observed that children with cancer consume less sodium after an average of 12 months of treatment compared to the amount after diagnosis (115).

### 4.5 Protein

Data regarding protein intake in children during cancer therapy are contradictory. Galati PC et al. and Tan et al. showed lower protein intake in children with cancer compared to control group (111, 114). Delbecque-Boussard et al. confirmed this, however in that study protein intake in children with cancer exceeded the recommended level (42). Skolin et al. demonstrated that protein consumption during hospital stays was below recommendations (116). Tah et al. indicated that children with hematology malignancies eat more protein than children with solid tumors (20). Soliman Bahgat et al. observed no difference in protein consumption between diagnose and after 3 months of treatment (110).

### 4.6 Energy

Tah et al. observed that children with hematology malignancies eat more calories than children with solid tumors (20). Warris L.T. showed that children with ALL after four-day dexamethasone administration had significantly increased energy, fat and saturated fatty acids intake (117). Gibson et al. also observed increased energy intake during steroid therapy (10). Delbecque-Boussard L. et al. observed reduced energy intake in children with ALL on day 1 and 22 of treatment, but there were no differences on days 36 and 71 compared with control group (42). Rohr et al. observed that children with ALL has 30% increased energy intake during induction compared to diagnosis and decreased energy intake during maintenance (113). In contrast, Tan et al. observed that children with ALL and AML had significantly lower intake of energy compared to control healthy children (114).

### 4.7 Sugar and carbohydrates

Cohen et al. observed that almost half of children with cancer exceeded recommendation for sugar (108). Moreover, patients had an increased desire to eat foods containing carbohydrates (108). The preference for carbohydrate-based meals was also confirmed by Skolin et al. (112). Galati et al. also observed high sugar consumption in children with cancer (111). Skolin et al. indicated that sucrose intake was higher than in healthy Swedish children (118).

Detailed information about the above studies are provided in **Table 3**.

**Table 3:** Summary of studies assessing the quality of diet and eating habits of children with cancer.

Author, year, reference	Type of cancer	Patients number (n)	Age (mean +/- SD	Control group	Assessment method and time	Assessed nutrients	Outcomes
Cohen et al., 2021 (108)	Parents of children with: ALL (n=33), AML (n=1), brain tumor (n=4), HL (n=4), NHL (n=4), NBL (n=2), osteosarcoma (n=1), soft tissue sarcoma (n=1), WT (n=4), other (n=1)	N = 64	8 (± SD 4,47)	–	M: 24-h dietary recall, questionnaire T: unspecified	Energy, protein, saturated fats, sugar, sodium, fiber, food groups, vitamins: B1, B2, B3, B6, B12, A, C; Folate, magnesium, calcium, phosphorus, iron, zinc, selenium, iodine, sodium, potassium	• Children did not consume adequate amount of vegetable (94% of children), fruit (77%), milk/alternatives (75%) • Half of consumed vegetable were classified as chips/fries) • 49% of children had sugar intake above the recommended level • 65% of children had sodium intake above the recommended level • Most children (95%) eat enough protein • 61% of children did not meet fiber recommendations
Warris et al., 2017 (117)	ALL	N=44	3-16	–	M: 4-days dietary diary	Energy, protein, fat, saturated fat, carbohydrate, sodium intake	• Increased intake of energy, protein, fat, saturated fat, carbohydrates and sodium between the 1st and 4th day of dexamethasone administration
Cohen et al., 2015 (11)	ALL (n=8), NBL (n=3), WT (WT) (n=3), BT (n=1) RMS (n=1)	N=18	8,5 (+/- SD 2,71)	N=18 age and sex-matched recruited from Sydney-based community organizations	M: Semi structured telephone interviews with parents T: > 5 and <13 years after treatment completion	Eating habits	Compared to eating habits before diagnosis, children after cancer treatment: • eats less fruit and vegetables • eats more “junk food” • eats larger portions Continuation of bad eating habits that appeared during cancer treatment. During cancer treatment: • decreased consumption of fruits and vegetables • increase in preferences for carbohydrate-based foods and savory biscuit • increased consumption of “junk food”
Soliman Bahgat et al., 2013 (110)	Leukemia (n=28), lymphoma (n=14), bone tumors (n=7), tumors of the nervous system (n=7), soft tissue tumors (n=4)	N=60	3-15	–	M: Structured interview sheet, 24 hours recall method T: 3 months after diagnosis	Energy, protein, calcium, iron, vitamin A and C, Number of meals per day, snacks	after 3 months of treatment: • the percentage of children eating snacks increased (73,33% vs 43,33%) • increase in the number of meals per day • the percentage of children eating meat decreased (50% vs 21,67%) • no significant differences between intake from specific food groups compared to the initial contact • no significant differences in consumption of energy, proteins, calcium, iron, vitamin A and C compared to initial contact
Tan et al., 2013 (114)	ALL (n=43), AML (n=10)	N=53	3-12	matched for sex, age (+/- SD 6 months) ethnicity with healthy children	M: 3-day food records T: During induction or consolidation phase	Energy, carbohydrate, protein, fat, calcium iron, vitamin A, B1, B2, B3, C	• Children with cancer had significantly lower intake of energy and protein compared to control group • Calcium intake in children with cancer was below the RDI
Fuemmeler et al., 2012 (59)	ALL, lymphoma	N=15	10,3	Healthy individuals in community, matched age, race, sex	M: 2-day food diary (1 weekday and 1 weekend day) T: Baseline, 6 and 12 months after diagnosis	Energy (kcal), % energy from protein, fat, carbohydrates, calcium, added sugar, food groups (serving size)	• Consumption of fruits, vegetables, dairy products below USDA recommendation • No significant differences in intake between study and control group
Gibson et al., 2012 (10)	ALL (n=1), relapsed ALL (n=2), NHL (n=2), HL (n=1), WT (n=2), relapsed WT (1), BT (n=1), relapsed AML (n=1), primitive neuroectodermal tumor (n=1)	N=13	4-12	–	M: 2 visual techniques: • phot graphs and drawings contained in albu • in-depth interviews with parents T: children at various stages of treatment: start (n= 6), middle (n=5), end (n=2)	Food habits and food preferences	• increased preference for savory food and strong flavors food • increased consumption during steroids • children did not want to eat hospital food, parents often had to look for takeaway food
Tah et al., 2012 (20)	Hematological malignancies (n=37), solid tumors (n=37)	n= 74	3-15	–	M: 3-day food record (2 weekdays and 1 weekend) T: induction (43,2%), consolidation (45,9%), maintance (5,4%), relapsed protocol (5,4%)	Energy, protein, carbohydrate, fat, calcium, iron, vitamin: A, B1, B2, B3, C	• Higher energy, protein, carbohydrate, vitamin A, and niacin intake in children with hematological malignancies • Higher percentage of patients with solid tumors had energy intake below the recommendations, compared to children with hematologic malignancies
Galati et al., 2011 (111)	Solid tumors (n=8), nonsolid tumor (n=8)	N=16	6-15 (11,05 ± SD 2,67)	N=19	M: interview recall method, T: during chemotherapy	Energy, carbohydrate, lipid, protein, fiber, calcium, iron, zinc, potassium, magnesium, phosphorus, vitamin: B1, B2, B3, B12, C; food groups	• No differences in the consumption of carbohydrates and fats between the study and control group • Higher protein and potassium intake in study group • Higher zinc, phosphorus, riboflavin, vitamin B12 intake in control group • Children with cancer had a higher intake of meat compared to the recommended amount • Low consumption of milk and dairy products, cereals, vegetables and high sugar consumption in both groups
Rohr et al., 2006 (113)	ALL	N=45	5 (+/- SD 2,6)	–	M: 24-h dietary recall T: Induction and reinduction	Energy, protein, zinc, copper	• 30% increased calorie intake during induction and reinduction compared to diagnosis • Increased desire to eat rice with beans, meat, bread, pasta • Decrease in energy and nutrient intake during the maintenance • Decrease in copper concentrations at the beginning of the study, and no changes during follow up • No significant changes in zinc concentration over the study period
Skolin et al., 2006 (119)	Leukemia (n=9), solid tumor (n=6), lymphoma (n=5), CNS tumor (n=2)	N=22	2-17 (madian=8)	Age and sex matched children to group of patients participating in taste acuity test (n=10)	semi-structured interviews time: 4 (1–12) months from initiation of chemotherapy to interview	Food preferences, taste acuity test	• Patient-reported food preferences: pancakes, pasta, potato dishes, taco shells, rice, salty snacks • Parent-reported preferences: salty foods, spicy and sour foods such as tomato soup, pickles and olives • Foods avoided by patients: red meat, hot dogs, and chicken (38% of children), sweets (29%), chocolate (10%) • Poor acceptance of commercial energy-dense drinks • Taste test: patients had a higher bitter taste threshold and made more mistakes in taste recognition compared to the control group
Skolin et al., 2001 (112)	CNS tumor (n=4), ALL (n=4), LCH (n=1), HL (n=2)	N=11	2-15 (median=7)	–	M: semi structured retrospective interviews with parents, who reported their child’s dietary habits at the start of treatment T: Since initiation of chemotherapy: 3 weeks – 1 year	Food preferences	• Carbohydrate-based dishes, macaroni, fried chicken, fast food, broccoli, and the avoidance of meat • Aversion to hospital food • More than 50% of parents participating in the study noticed that their child’s dietary choices changed after starting chemotherapy • Daily energy intake ranged from 20% to 80% of the RDI
Skolin et al., 2001 (116)	CNS tumor (4), ALL (4), LCH (1), HL (2)	N=11	2-15 (median = 7)	–	M: 21-d dietary food record T: Day 0 of chemotherapy	Energy, protein, fat, carbohydrate	• The average energy and nutrient intake during the hospital stay was 63% of RDI, with nutritional support 88% of RDI • energy, protein, carbohydrate intake during “hospital days” below recommendations
Delbecque-Boussard et al., 1997 (42)	ALL	n=15	mean = 6.2	N=15 healthy children matched for sex and age	24-h dietary recall Time: Days 22, 36, 71 of chemotherapy	Energy, protein, fat, carbohydrate	• Reduced energy intake in cancer patients on day 1 and 22, but no differences on days 36 and 71 compared with control group • On day 1 60% of caner children consumed < 80% energy of the French RDA • Protein intake was above French RDA recommendation in all cancer children • Significant differences in carbohydrates intake from day 1 to 71 • No significant differences in fat and protein intake from day 1 to 71
Skolin et al., 1997 (118)	ALL (n=3), CNS tumor (n=4), sarcoma (n=3), lymphoma (n=3), WT (n=1)	N=14	5-16 (median = 10)	–	M: 21-d dietary food record T: Day 1 of chemotherapy	energy, protein, fat and carbohydrate	• The average energy and nutrient intake of children with cancer during the hospital stay was 63% of the Swedish Nutrition recommendations • The average daily energy intake during staying at home remained below 77% of the Swedish Nutrition recommendations • Preferred products: bread, vegetables, ice cream, biscuits, fresh fruits • After few days in hospital, preferred products: bread, butter, dairy products • Sucrose intake in children with cancer was higher than in healthy Swedish children

ALL, acute lymphoblastic leukemia; M, method; T, time; USDA, United States Department of Agriculture; BT, brain tumor; NBL, neuroblastoma; RMS, rhabdomyosarcoma; HL, Hodgkin lymphoma; NHL, Non-Hodgkin Lymphoma; CNS, central nervous system; RDI, Recommended Daily Intake; RDA, Recommended Dietary Allowances; WS, Wilms Tumor; LCH, Langerhans cell histiocytosis; AML, Acute myeloid leukemia; SD, standard deviation.

Vitamin D deficiency is observed in AYA cancer patients, especially in those with ALL and testicular cancer. In addition, calcium, folate and iron deficiency is also noticeable in this group of patients (120).

### 4.8 Interventions to improve nutritional status and diet quality in children with cancer

The above section indicates the need for dietary intervention in children with cancer. Bélanger et al. conducted a study in group of children with cancer and (n=62). Participants were enrolled to study 4 to 12 weeks after diagnosis. The intervention lasted for a year and consisted of every 2 months check-ups with a registered dietitian. Most children and parents had high participation - 50,8%, and high engagement - 56,4%. Patients with refractory disease or relapse had lower intervention completion rates. Patients who completed the intervention had lower sodium intake compared to baseline intake (n=21) (115). Napartuk M. et al. conducted a study also assessing changes in diet after a one-year dietary intervention and changes in anthropometric measurements and cardiometabolic profile in pediatric patients with cancer (n=36). They observed improvement in diet quality reflected by the Diet Quality Index (5,22 ± 9,95, p = 0,003), z-scores for weight (p = 0,019), BMI (0,50 ± 0,88, p = 0,002), mean levels of HDL-C (0,27 ± 0,37 mmol/L, p = 0,002) and 25-hydroxy vitamin D (14,5 ± 28,1 mmol/L, p = 0,03) (107). Zhang FF. et al. conducted a study on early lifestyle intervention in ALL survivors. The intervention lasted 12 weeks, and 15 participants and their parents were qualified to participate. After 12 weeks of intervention, reduced pressure to eat (p = 0,03), increased milk consumption (0,54 serving/d, 0,02 to 1,07; p = 0,04) and percent of calories from protein (2,54%, 0,22 to 4,87%; p = 0,04) and lower potato consumption (−0,16 serving/d, -0,30 to −0,03; p = 0,02) (121). Viscardi S. et al. also conducted a nutritional intervention in children with cancer and their parents (n=20), who received 6 educational sessions. After educational intervention, authors observed reduction in consumption of unhealthy foods (sugar drinks and candy (p>0,005)) and significant increase in consumption of healthy food (water, vegetables, fruits p (<0,005)) (122).

## 5 AYA cancer patients

The adolescent and young adult cancer population is a group of patients aged 15-39 characterized by gaps and challenges in terms of cancer diagnosis, participation in clinical trials, access to research and improving cure rates (123). Patients within this age bracket are in a phase of intensify personal development, and a cancer diagnosis is associated with psychological burden, financial challenges and fertility issues (124). The most common cancers in this group of patients are breast cancer, melanoma, colorectal cancer, sarcoma, and ALL (125). More than 1 million new cases of cancer are diagnosed each year in the AYA population worldwide (123). Despite the increasing interest in this group of patients, there is a disproportion in the morbidity and cure rates of AYA patients and children and the elderly (124). In the case of ALL, mortality in children has decreased significantly since 1970, while in the AYA population the reduction was 30-35% lower than in children (123). In AYA patients 5-year relative survival of breast cancer is worse than in older women (125). Diagnosing cancer is also challenging because the symptoms are non-specific and differ from those seen in adults, which can prolong the diagnostic process (126). Additionally, AYA patients show lower levels of adherence to therapy compared to older patients and consider breaking off therapy (127).

AYA cancer patients are particularly vulnerable to depression (128). This is related, among other things, to negative changes in their current lives, such as temporary resignation from work or education, disturbed family and peer relationships, as well as being increasingly dependent on other people (129, 130). AYA survivors are also at higher risk of depression and other mental disorders (131, 132). Bacling N.V. et al. conducted a questionnaire study in group of AYA survivors (n=639) on mental health disorders (133). They observed that AYA survivors had more severe depression [incidence rate ratio=1,42, 95% confidence interval (CI)=1,09 to 1,84, P<0,001] and anxiety (incidence rate ratio=1,85, 95% CI=1,55 to 2,21, P<0,001). Moreover, they were more likely to use psychotherapy (odds ratio=1,91, 95% CI=1,16 to 3,17, P<0,005) and mental health medications (odds ratio=1,89, 95% CI=1,15 to 3,11, P<0,005) (133). Rosgen B.K. et al. observed that AYA survivors have higher risk of anxiety, depression and trauma- stressor-related disorders (134).

Another challenge for the AYA cancer patient is increased treatment toxicity (135). They experience more severe toxicities than children when treated with identical chemotherapy regimens (135). Gupta A. conducted a study in which he analyzed health care outcomes in pediatrics (ages 10-14) and AYA patients (ages 15-39) in a total of 4046 patients. It was observed that compared to children, AYA patients had increased toxicities in almost every organ system. Moreover AYA patients had a significantly higher incidence of intensive care unit stay but there were no differences in median hospital stay nor mortality (136). One reason is altered drug metabolism in AYA patients and children (135). Children show an oral dexamethasone and methotrexate clearance rate that is twice as fast compared to AYA cancer patients (137). These age-related distinctions are associated with an elevated incidence of osteonecrosis (ON) and mucositis toxicity in AYAs compared to younger children with ALL (135). During puberty, the body composition of patients changes, the fat tissue content increases in girls, the muscle tissue increases, and the fat tissue decreases in boys. This may change drug distribution. Additionally, at this age, hormonal changes occur that may alter the action of drug-metabolizing enzymes (135). The most common side effects occurring in AYA patients include vincristine-induced neuropathy (VIN), osteonecrosis, myelosuppression/infection, and hematopoietic stem cell transplant complications (135).

## 6 Discussion

According to current scientific knowledge, in children with cancer both undernutrition and overnutrition are associated with increased treatment toxicity, worse OS and EFS rates, worse physical, emotional and social functioning. Cancer is often associated with underweight, but due to increased prevalence of overweight in children worldwide, more and more children begin cancer treatment with initially excessive body weight. Malnutrition and overnutrition may occur both at the beginning and during treatment, so regular monitoring is necessary. Nevertheless, our review revealed that measuring only body weight may be insufficient. Available research indicates that body composition in children with cancer changes during oncological treatment - lean body mass decreases or remains low while fat mass increases. The greatest changes occur in the first months of cancer treatment. This affects chemotherapy volume distribution, metabolism and clearance. There is a great need for research on changes in body composition and related drug pharmacokinetics in children with cancer.

There are many methods of assessing body composition, among which an easily available and cost-effective is the measurement of mid-upper arm circumference. This method accurately reflects the level of lean body mass. Measurement of body composition allows also for the diagnosis of sarcopenia, the occurrence of which in cancer patients is associated with higher risk of infection, poorer treatment tolerance and more common dose-limiting toxicities. It is necessary to develop standards of practice in the assessment of body composition and nutritional status in this group of patients.

Another serious problem among pediatric cancer patients is the low quality of diet both during and after oncological treatment. Due to changes in taste and smells, nausea, vomiting and diarrhea, the intake of macro- and microelements in recommended amounts is difficult. In this review, we observed that consumption of milk, dairy products, vegetables and fruits in children with cancer is too low, while the consumption of sugar often exceeds recommendations. Nutritional support should start at the time of cancer diagnosis and be provided during and after treatment.

## Author contributions

MS: Conceptualization, Visualization, Writing – original draft, Writing – review & editing. SS: Conceptualization, Visualization, Writing – original draft, Writing – review & editing.
